# Growth mixture models: a case example of the longitudinal analysis of patient‐reported outcomes data captured by a clinical registry

**DOI:** 10.1186/s12874-021-01276-z

**Published:** 2021-04-21

**Authors:** Jae-Yung Kwon, Richard Sawatzky, Jennifer Baumbusch, Sandra Lauck, Pamela A. Ratner

**Affiliations:** 1 School of Nursing, University of British Columbia, Vancouver, Canada; 2 School of Nursing, Trinity Western University, 22500 University Drive, V2Y 1Y1 Langley, BC Canada; 3 Evaluation and Outcome Sciences, Providence Health Care Research Institute, Vancouver, Canada; 4 Sahlgrenska Academy, University of Gothenburg, Gothenburg, Sweden; 5 St. Paul’s Hospital, Vancouver, Canada; 6 Department of Education and Counselling Psychology, and Special Education, Faculty of Education, University of British Columbia, Vancouver, Canada

**Keywords:** Growth mixture modelling, Patient-reported outcomes, Clinical registry

## Abstract

**Background:**

An assumption in many analyses of longitudinal patient-reported outcome (PRO) data is that there is a single population following a single health trajectory. One approach that may help researchers move beyond this traditional assumption, with its inherent limitations, is growth mixture modelling (GMM), which can identify and assess multiple unobserved trajectories of patients’ health outcomes. We describe the process that was undertaken for a GMM analysis of longitudinal PRO data captured by a clinical registry for outpatients with atrial fibrillation (AF).

**Methods:**

This expository paper describes the modelling approach and some methodological issues that require particular attention, including (a) determining the metric of time, (b) specifying the GMMs, and (c) including predictors of membership in the identified latent classes (groups or subtypes of patients with distinct trajectories). An example is provided of a longitudinal analysis of PRO data (patients’ responses to the Atrial Fibrillation Effect on QualiTy-of-Life (AFEQT) Questionnaire) collected between 2008 and 2016 for a population-based cardiac registry and deterministically linked with administrative health data.

**Results:**

In determining the metric of time, multiple processes were required to ensure that “time” accounted for both the frequency and timing of the measurement occurrences in light of the variability in both the number of measures taken and the intervals between those measures. In specifying the GMM, convergence issues, a common problem that results in unreliable model estimates, required constrained parameter exploration techniques. For the identification of predictors of the latent classes, the 3-step (stepwise) approach was selected such that the addition of predictor variables did not change class membership itself.

**Conclusions:**

GMM can be a valuable tool for classifying multiple unique PRO trajectories that have previously been unobserved in real-world applications; however, their use requires substantial transparency regarding the processes underlying model building as they can directly affect the results and therefore their interpretation.

## Background

A common challenge facing health researchers is how to examine and respond to the variability in patient-reported outcome (PRO) responses to treatment over time, particularly in heterogeneous clinical populations with complex real-world data. For example, unobserved subgroups of patients may exist within the clinical population, which may exhibit variability in their treatment responses and have different health trajectories. This heterogeneity is typically masked when group means are considered. Identification of multiple PRO trajectories and their risk factors could have significant clinical implications, which (if better understood) could inform tailored interventions and patient education strategies (i.e., based on the characteristics of, and appropriate for, the subgroups). One promising approach that can be used to identify multiple unique PRO trajectories in heterogeneous clinical populations is Growth mixture modelling (GMM; a.k.a. latent variable mixture models), which is suitable when subgroups of patients with different trajectories are expected and grouping variables are not known *a priori*. This is an expository paper that demonstrates the use of GMM and the steps that were taken in an analysis of longitudinal PRO data captured by a clinical registry for outpatients with atrial fibrillation (AF).

There are many approaches available for the modelling of patients’ trajectories of change in their PRO or health status (e.g., multilevel modelling (MLM) and latent growth modelling (LGM)). However, an important limitation of these techniques is that relevant group differences in the clinical population must be specified, *a priori* (e.g., demographic or clinical differences). Yet, there may be unobserved subgroups of trajectories associated with differences that are not known and thus not possible to specify *a priori* (e.g., as defined by various demographic and clinical differences, and interactions among them). More important, these models take a variable-centred approach, which presumes that people are a medium through which predictor variables affect outcomes and assume that trajectories are similarly experienced by all individuals (an assumption that underlies most clinical research) [1]. While these types of analyses provide a single set of parameters that may reveal general health improvements over time (e.g., whether a treatment, on average, works for most people), they can mask subgroups of trajectories within a clinical population, and therefore may not be representative of all patients [2]. Since clinical populations typically include diverse people with different demographic and clinical factors affecting their health trajectories, it is unlikely that all patients would be adequately represented by a single health trajectory. Accordingly, important subgroups of patients may emerge with distinct patterns of change not known *a priori* if an appropriate analytical approach is applied. Thus, flexible modelling approaches are needed that could provide more nuanced pictures of subgroups of patients within heterogeneous clinical populations.

**Fig. 1:**
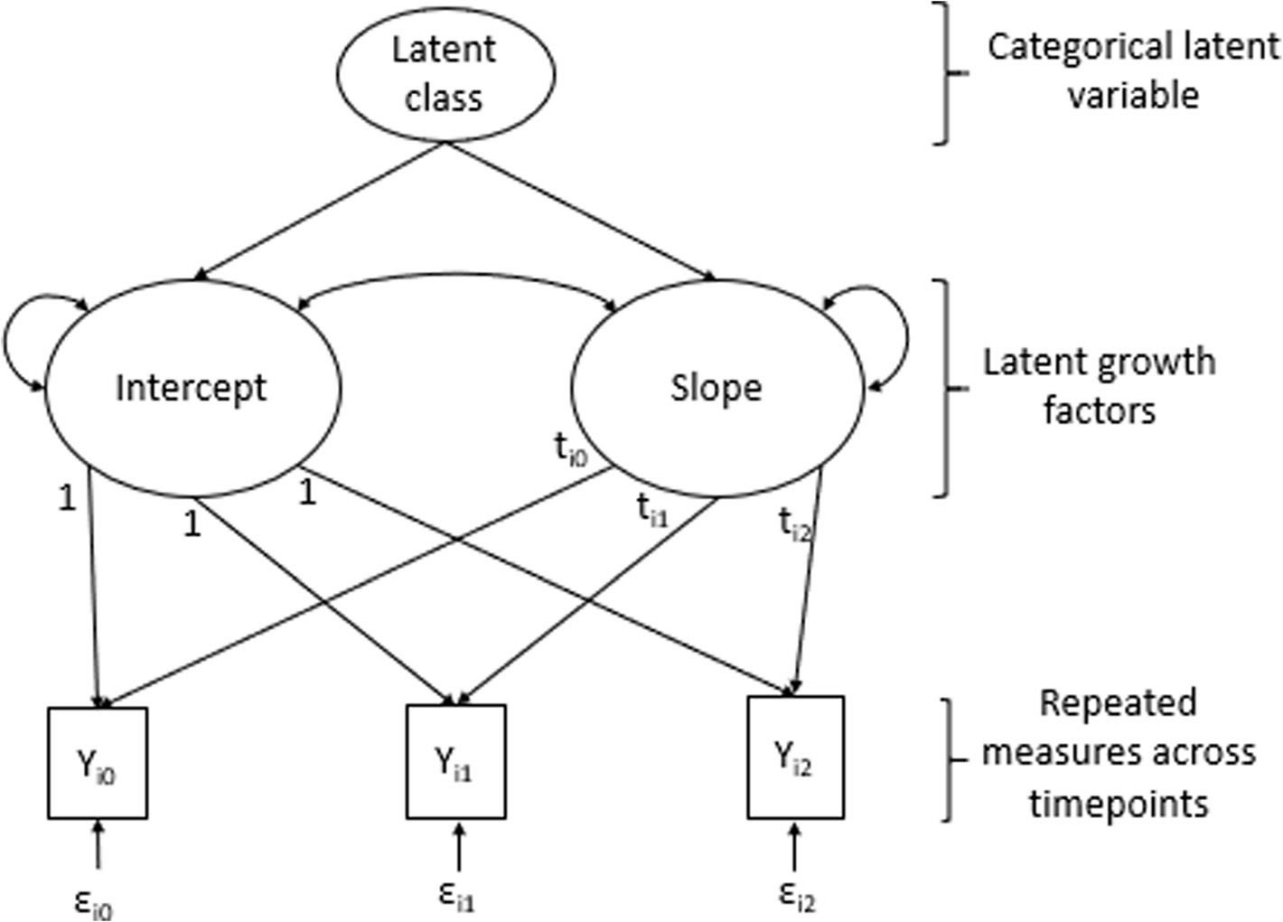
Growth mixture model with three continuous outcomes

GMM is considered to be a person-centred method because it is predicated on the assumption that people are the agents that affect the outcomes of interest with predictor variables deemed to be properties of those people, and also on the assumption that trajectories are different across individuals or subsets of individuals [1]. GMM works by assigning individuals who share similar patterns of scores into unobserved subgroups called latent classes. These latent classes are based on probabilities in which each individual receives fractional membership in all classes to reflect varying degrees of precision in their classification [3]. GMM extends the LGM approach because it incorporates a categorical latent variable, which represents mixtures of subgroups where membership is not known *a priori* but is inferred from the data. In this way, latent classes represent subgroups of individuals who follow approximately the same trajectory. For example, patients can be classified into different latent subgroups with different trajectories of change with their own initial status (intercept) and rate of change (slope). GMM also extends the MLM approach because the slope loadings are constrained to allow for nesting of time observations within individuals, which allows each individual’s slope to represent the unique times at which their assessments were completed (see Fig. 1).

The Yi_0_ – Yi_2_ represents the outcomes at the varying time points for individual i. These variables are used as indicators of latent variables that represent different aspects of individuals’ change known as latent growth factors. There are two latent variables (sometimes called random coefficients). The first is the latent intercept, which represents the level of the outcome when time is zero (baseline), and thus the intercept factor loadings are all fixed to one as a constant. The second is the latent slope, which represents the change in the outcome over time. The slope loadings are fixed to reflect the time since initial baseline status. The slope loadings are labelled as t_i0_ – t_i2_ to reflect the individually varying times. In a basic GMM, each individual has an estimated intercept and slope, which are allowed to vary across individuals. This variability across individuals is estimated as the variance of the latent intercept and slope and is depicted as a double-headed arrow that points to the same variable. The intercept and slope are shown to covary and modelled in the figure to show how individuals’ start or baseline values relate to their rate of change. The latent variables also have means to reflect the average of all individuals’ intercepts and slopes. In addition, individuals have their own deviations from those means at each time point known as residual/error variances, which are depicted as ε_i0_-ε_i2_.

While GMM is a good modelling choice when subgroups of patients with different trajectories are expected and grouping variables are not known *a priori*, its flexibility comes at a price because all relations between observed and latent variables have to be specified [4]. We offer guidelines and recommendations for (a) determining the metric of time, (b) specifying the GMM, and (c) including predictors of the latent classes.

## Methods

We present lessons learned from our experience in analysing repeated PROs collected for a population-based clinical registry of outpatients with AF. More detail of the methods employed is provided elsewhere [5]. In brief, the study relied upon data derived from a retrospective cohort of outpatients who had been referred, between 2008 and 2016, to five AF clinics (for new-onset and persistent AF in need of complex anticoagulation/ablation therapy) in a province in western Canada and who provided data for the clinical registry. The AF clinics’ registry database [6] was deterministically linked to administrative health data, including Consolidation files (a central demographics file) [7], Discharge Abstracts Database (Hospital Separations) (data on discharges, transfers and deaths of in-patients and day surgery patients from acute care hospitals in the province) [8], the Medical Services Plan (public insurer) payment files (data on medically necessary services provided by fee-for-service practitioners to individuals covered by the provincial universal insurance program) [9], PharmaNet files (data on all prescriptions for drugs and medical supplies dispensed from community pharmacies and prescriptions dispensed from hospital outpatient pharmacies for patient use at home) [10], and Vital Statistics – Deaths (includes all deaths registered in the province) [11].

The primary PRO measure was the Atrial Fibrillation Effect on QualiTy-of-Life (AFEQT) questionnaire [12], which is composed of 20 items that assess four domains: symptoms (4 items), daily activities (8 items), treatment concerns (6 items), and treatment satisfaction (2 items). The analyses were conducted on the patients’ summary score that incorporates the responses of the first three domains. The collection of the AFEQT questionnaire varied throughout the follow-up period (the enrolled patients had up to 10 visits to the clinics over a maximum 5-year period) depending on the complexity of the patients’ management, clinic wait times, and individual patients’ decisions about whether to complete the questionnaire at each visit. This paper focuses on some of the challenges encountered while using GMM with longitudinal PRO data stored in a clinical registry.

## Results

### Determining the metric of time

**Fig. 2:**
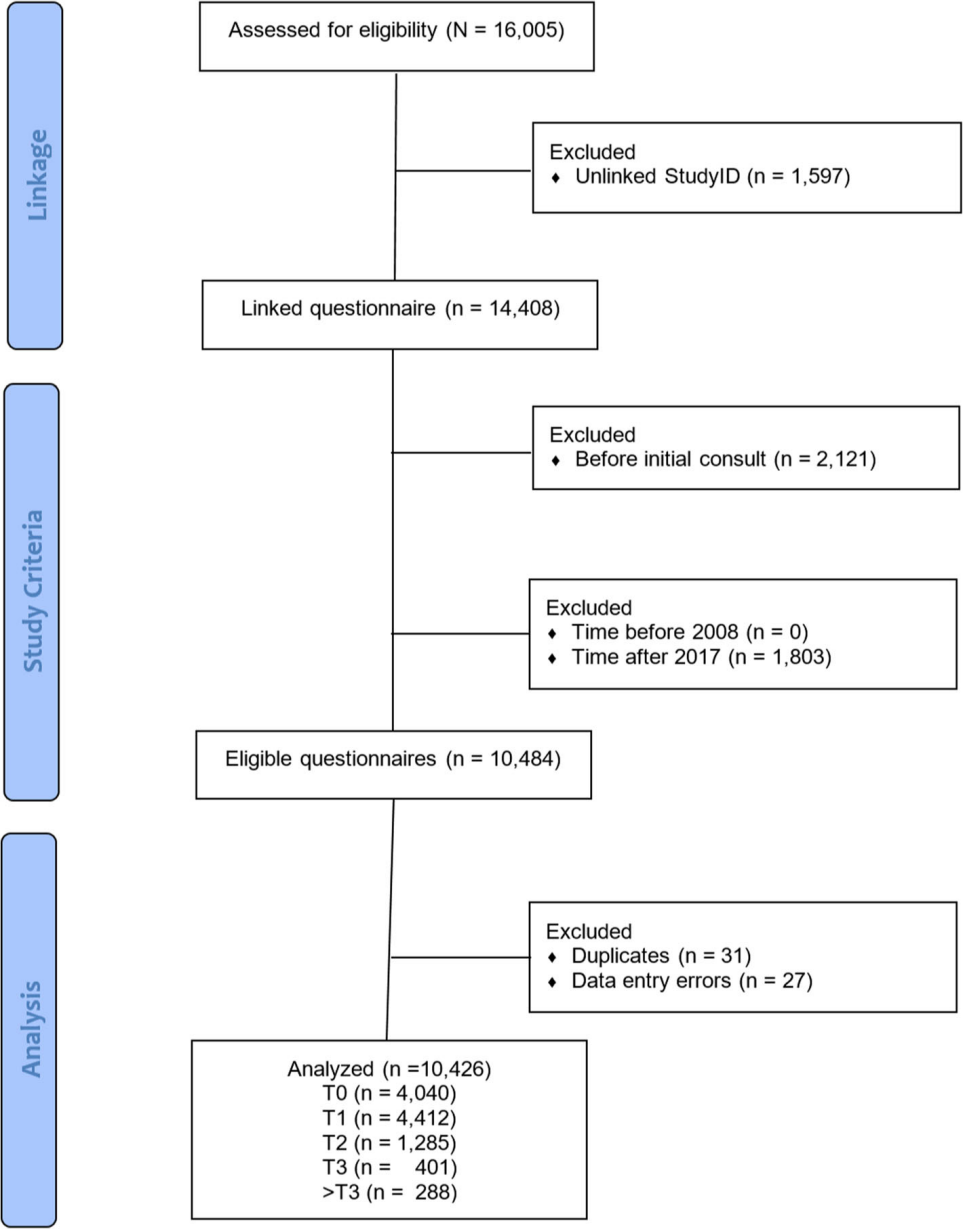
Flowchart of the eligible clinical registry cohort’s completed AFEQT questionnaires available for analysis. *Note. “*n” refers to number of AFEQT questionnaires completed (not Study IDs or individuals)

The first step in preparing for the longitudinal analysis was to use various graphical techniques including flowcharts, time series and smooth curve plots to get an overview of the PRO data collection. Because the patients could have more than one completed questionnaire in the registry, each Study ID could have more than one entry. Thus, the flowchart describes the inclusion of unique questionnaires, not unique individuals (see Fig. 2).

While GMM is a good modelling choice when subgroups of patients with different trajectories are expected and grouping variables are not known *a priori*, its flexibility comes at a price because all relations between observed and latent variables have to be specified [4]. We offer guidelines and recommendations for (a) determining the metric of time, (b) specifying the GMM, and (c) including predictors of the latent classes.

The AFEQT questionnaire dataset (*n* = 16,005 completed questionnaires) was linked to the eligible cohort of patients based on their unique identification number (StudyID), resulting in 14,408 questionnaires (some questionnaires could not be linked to a patient (*n* = 1,597)). After excluding questionnaires based on the inclusion criteria (must not have been completed before the initial consultation (*n* = 2,121) or after 2017 (*n* = 1,803)), and removing any duplicates (*n* = 31) or questionnaires with data entry errors (*n* = 27), 10,426 PRO questionnaires were available for analysis.

**Fig. 3:**
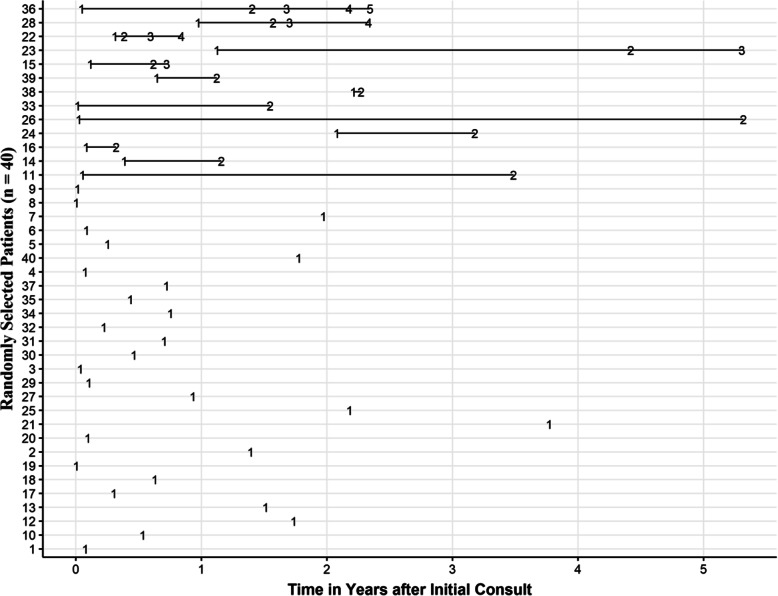
Number of PRO questionnaires completed by 40 randomly selected patients by time (in years)

To determine the time metric to be used to fit the model, we plotted the individual time intervals of the repeated PRO measures for 40 randomly selected patients (see Fig. 3).

The uppermost lines in Fig. 3 show 13 of the 40 patients who had two or more questionnaires completed; the figure reveals that the remaining patients completed only one questionnaire at varying times, including from the time of their initial consultation to more than 3 years after the initial consultation. These graphs represent an informative picture of the timing of the repeated measures and provide a view that is not readily apparent from conventional descriptive statistics of study data. The traditional approach to modelling time is either to use the number of follow-up visits (tied to the frequency of PRO measures completed) or as the time intervals between the initial measurement and subsequent assessments (tied to the dates when the PRO measures were completed). However, Fig. 3 shows that the first PRO measure completed was not directly tied to the initial consultation and the intervals between the PRO measure completions were not of equal duration (e.g., every 6 months).

**Fig. 4:**
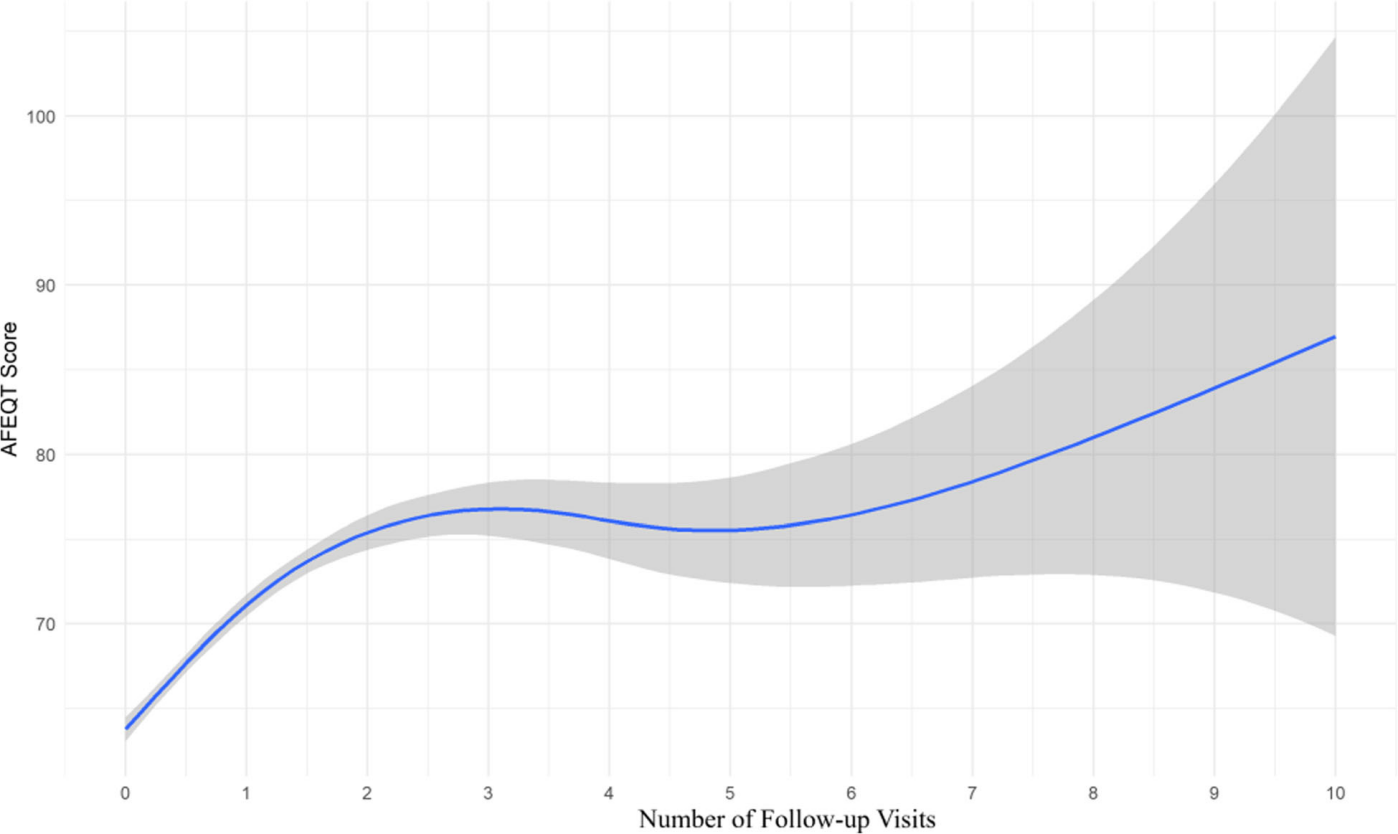
Smooth curve of AFEQT questionnaire scores by number of follow-up visits

**Fig. 5:**
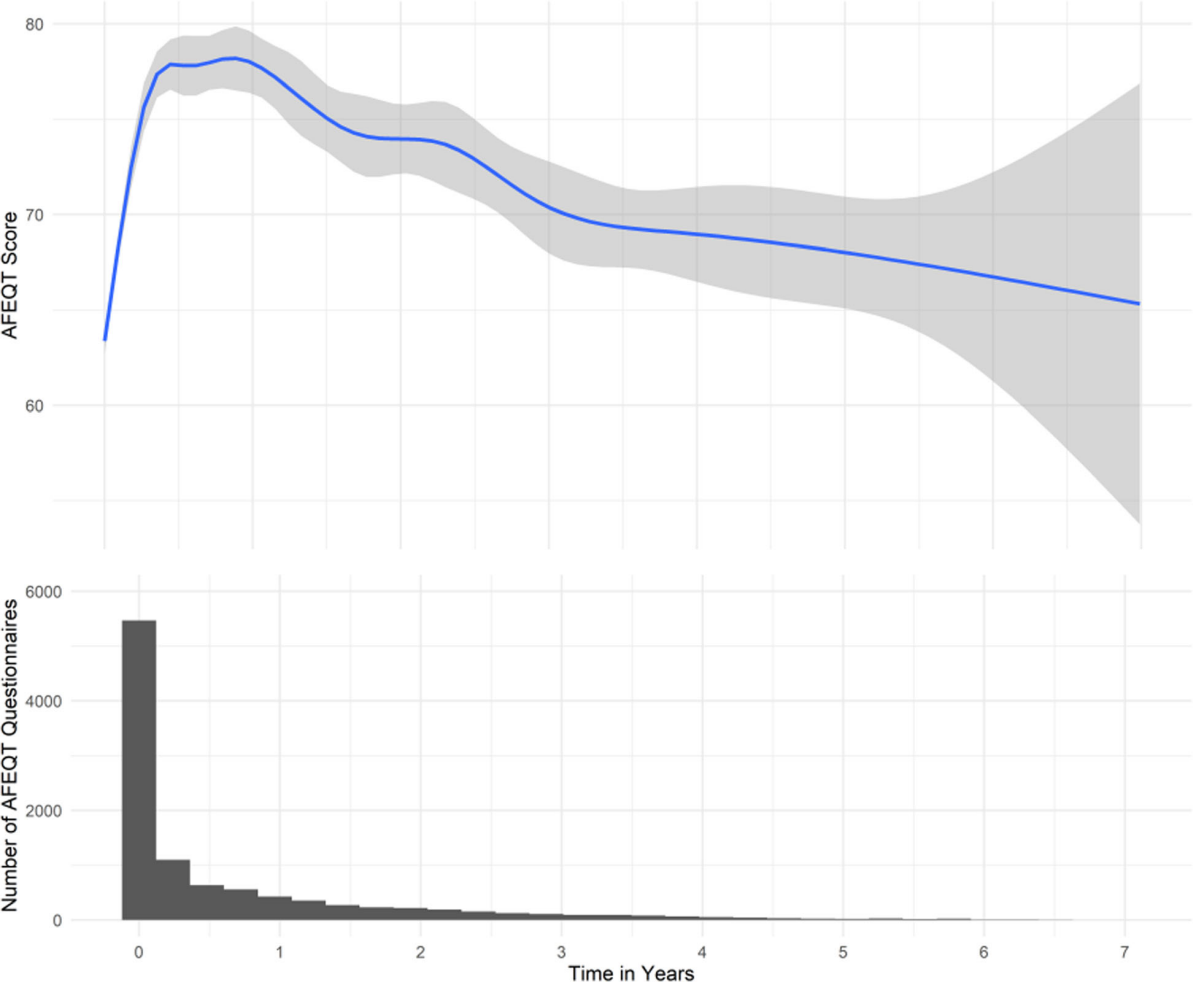
Smooth curve of AFEQT questionnaire scores with the number of questionnaires administered, over time in years

To further examine each of the time metrics, we plotted the trend of the AFEQT summary scores by the number of follow-up visits and by time in years (see Figs. 4 and 5, respectively).

There was a general upward trend in the PRO scores (blue line) over the number of follow-up visits with the 95 % confidence intervals (shaded area) widening as the data points became increasingly sparse.

However, when the PRO scores were plotted against continuous time in years, the opposite trend was observed: there was a rapid improvement in scores until an inflection point at about one-year of follow-up with a gradual decrease over time from then on. There are several possible explanations for these marked differences, including the individual variation in length of time between each visit and the variation in the number of visits for each person. In longitudinal analyses of PROs captured in clinical registries, it may be difficult to completely separate the number of follow-up visits from the time elapsed. For example, changes in the PRO questionnaire scores may have been influenced by both clinic-related processes, when the patients followed a certain treatment protocol, and time-related processes, when the sicker patients may have been followed by the clinic for a longer period of time. Thus, the metric of time had to account for the individually varying times of observation, which refers to the situation in which the patients were followed at different intervals (e.g., 3 weeks→10 weeks→4 months), rather than evenly spaced intervals (e.g., every 6 months). To align what we were observing in practice to the method of analysis, the model had to represent both the variability in the *frequency* and the *timing* of the measurement occurrences.

### Specifying growth mixture models

To begin an initial examination of a GMM, a series of models are specified and subsequently estimated. According to Gilthorpe et al. [13], the selection of a suitable GMM with the “correct” number of latent classes is heavily influenced by the method used to parameterise the random effects within the model. For example, one approach is to freely estimate the growth factor variances and covariances for each latent class (referred to as unrestricted GMM). In contrast, an extreme form of model parsimony is to constrain all the growth factor variances to be equal, referred to as latent class growth analysis (LCGA) [49]. To identify the best baseline model prior to GMM specification, we conducted several single-group LCGAs using four time points. These included intercept-only, linear, and quadratic models (see Table 1). The intercept-only model represented the initial (baseline) levels of the AFEQT questionnaire scores, whereas the linear model included the linear change in the AFEQT questionnaire scores (i.e., a slope). For the quadratic model, an additional latent variable was added to the linear model to estimate a nonlinear pattern.

**Table 1:** Likelihood statistics for GMM models of change with four time points

	-2LL	df	AIC	BIC	SABIC
LCGA					
Intercept-only	-46,156.95	6	92,325.90	92,367.36	92,348.32
Linear	-46,065.15	9	92,148.29	92,210.52	92,181.92
Quadratic	-45,910.73	13	91,847.46	91,937.35	91,896.04
GMM – quadratic					
1-class	-45,910.73	13	91,846.46	91,937.35	91,896.04
2-class	-45,910.73	27	91,875.47	92,062.16	91,976.36

*Note*. *LCGA *Latent class growth analysis, *GMM *Growth mixture model, *LL *Log-likelihood, *df *degrees of freedom, *AIC *Akaike’s information criterion, *BIC *Bayesian information criterion, *SABIC *Sample size adjusted BIC

Because the slope loadings varied across individuals, traditional SEM goodness-of-fit statistics or the mixture model statistical comparison tests such as the Lo-Mendell-Rubin Test (LMRT) [14] and the Bootstrap Likelihood Ratio Test (BLRT) [15] were not available. Instead, the comparative fit of the models was primarily assessed using the Akaike’s information criterion (AIC), the Bayesian information criterion (BIC), and the sample size adjusted BIC (SABIC). Among the fit indices, the general principle of selecting the preferable GMM is to choose the lowest BIC and SABIC values (lower values indicate better model-data fit) because the associated model provides greater accuracy in identifying the latent classes compared with the model represented by the best AIC [16]. In Table 1, the BIC and SABIC for the LCGA models suggested that the quadratic model better fit the data than the intercept-only or linear model. This baseline quadratic model was used to estimate the GMM by freely estimating all the parameters (i.e., the latent means, variances/covariances, and residuals). However, the 2-class quadratic GMM resulted in a poorer model fit than the baseline 1-class model, which indicated that the model was not appropriate for the given data. Part of the issue in fitting the 2-class quadratic GMM may have been insufficient sample size, especially in the amount of data available for the last of the four time points because studies have shown that small samples can lead to convergence issues, improper solutions, and the inability to identify meaningful subgroups [3, 17]

What constitutes an “adequate” sample size is difficult to determine because it depends on the specification of the model, the distribution of the variables, the amount of missing data, and the strength of the relationships among the variables [18]; in general, large sample sizes (≥ 500) are often deemed most appropriate for complex mixture models [19]. In our dataset, the second follow-up visit (T2) met this criterion with a sample size of 1,285; there was a large reduction for the third visit (T3) with a sample size of 401. Although it is not unusual to have declining samples in longitudinal studies [20], due in part to early discharge from a clinic or through attrition, we narrowed our analysis to three time points (T0-T2) to ensure better identification of the trajectories and meaningful subgroups of patients.

Although we were limited to the linear GMM (due to having only three time points), many combinations of constraints were still possible (e.g., fixing the intercepts, slopes, residual variances or a combination of all three), which could have affected the selection and interpretation of the GMMs. A recent study has shown that placing certain constraints on variance parameters across classes or over time can strongly influence model performance [21]. To address the issue of model specification, we applied the three recommended GMM parameterisations of Gilthorpe et al. [13]: (a) unrestricted random effects, (b) restricted random effects (random intercepts only and no covariances), and (c) restricted random effects plus AR1 (an autoregressive structure). The unrestricted random effects model is specified to freely estimate all parameters, including the latent means of the intercept and the slope, variances, and covariances. However, such free estimation can lead to convergence issues and thus some constraints are typically applied. The restricted random effects model may aid convergence by constraining the slope variance to zero. In contrast, the restricted random effects plus AR1 is modelled because specifying constraints can lead to autocorrelation issues; this is a more parsimonious model. In other words, rather than assuming that each observation of the PRO scores was independent of the others, the autoregressive structure recognises that closely timed repeated measures are more strongly correlated than measures that are timed further apart. The final GMM (3-class restricted standard model) was selected based on the smallest information criteria (i.e., BIC and SABIC).

### Including predictors of latent classes

Once the final GMM was determined, we examined the predictors that explained the variability in the subgroups’ identified trajectories (the latent classes). There are two general approaches regarding how to include predictors or covariates and the outcomes of the latent classes in GMM: a one-step (joint model estimation) approach and a three-step (stepwise estimation) approach. The one-step approach uses a joint model that combines the latent class model with a latent class regression model such that the latent classes are conditioned on the covariates [22]. While the one-step approach may result in improved accuracy if the correct covariates are included (e.g., smaller standard errors), the most obvious disadvantage is that the inclusion of covariates may affect the type of classes found as well as class membership [23]. For example, both the latent class model and the latent class regression model need to be re-estimated each time a covariate is added. This may not only be impractical in most exploratory studies with many covariates but may cause the latent class variable to lose its meaning because it is no longer based on the original indicator variables [23].

To address this issue, the three-step approach was developed to independently evaluate the relationships between the latent classes and the predictor variables, such that the addition of predictor variables into the model does not change class membership itself [22, 23]. This approach involves first estimating the GMM using only latent class indicator variables (e.g., the AFEQT questionnaire scores) without covariates. In the second step, the most likely latent classes are created based on the posterior probabilities obtained in the prior step. In the third step, the latent classes are regressed on the predictor variables with multinomial logistic regression while adjusting for classification uncertainty in the second step [22]. To apply the three-step approach, we used the R3STEP method in Mplus version 8.3 [24] to conduct both bivariable and multivariable logistic regression analyses. The advantage of using the R3STEP is that the three-step procedure is implemented automatically rather than having to perform each step manually. The limitation of this approach is that the R3STEP does not allow for hierarchical (i.e., stepwise) regression models because model fit occurs only at the level of the GMM, which means that the fit indices will be the same regardless of the predictor variables entered. This is necessary to hold the class proportions fixed at the values identified when each predictor is entered into the model. In the case with GMM, the predictor variables explain complex relationships between both within-class variation as well as the probability of class membership. We then followed the variable selection approach recommended by Hosmer and Lemeshow [25] with modifications based on the identified health trajectories. For example, the univariate analysis for each of the variables compared one group, serving as a referent, to each of the other groups to identify predictors of latent class or subgroup membership.

## Discussion

One of the major issues in identifying and estimating our model was selecting an appropriate time metric because the interpretation of the parameters (i.e., the intercepts and slopes) and subsequently how the trajectories actually looked depended on this very choice. In longitudinal studies, and in registries that routinely collect PRO data in particular, individuals are often assessed at different time points and the number of assessments for each individual may vary, resulting in an unbalanced design [26]. Simulation studies have shown that ignoring individual differences in time points can lead to biased estimates in the baselines (intercepts) and rates of change (slopes) in the trajectories [27, 28]. In our study, we found that how we coded time could affect the results. For example, had we coded the time metric as the number of follow-up visits, the health trajectories would have shown subgroups of patients with rapid improvements over time. In contrast, if we had coded the time metric as a continuous variable in years elapsed to the last measurement, the health trajectories would have shown a subgroup of patients whose health status was apparently decreasing over time. However, it was obvious that ignoring the spacing of the observations would not have accurately represented the underlying processes of the clinical practice whereby the patients had different numbers of follow-up visits at varying time intervals. Studies have shown that the length of the follow-up period and the spacing in between time points affect the number of trajectories that can be found [29, 30]. Therefore, the usefulness of the model depends not only on transparently reporting the underlying time-related processes but also on how the time metric in the model is specified.

The next step was to include the covariates/predictors to predict class membership. While there is general agreement that incorporating covariates is beneficial in providing more accurate parameter estimates and recovering the correct number of classes [31, 32], there has been little consensus about when these covariates should be included. Some researchers argue that covariates of latent group membership should be included when deciding on the number of latent classes [33, 34], while others advocate that they be included only after the number of classes has been identified [35, 36]. This debate has played an important role in the development of many new analytic techniques in handling covariates, including variations of the 1-step (joint model estimation) approach that supports the former argument and the 3-step (stepwise estimation) approach that supports the latter. Although the 1-step approach provides more accurate parameter estimates when appropriate covariates are included, most researchers prefer using the 3-step approach for several reasons. The first is that the construction of a growth trajectory and examining how covariates affect these trajectories are often seen as two different steps in the analysis [37]. For example, the latent classes that we identified were based on the different trajectories (outcomes), and the covariates were based on individual characteristics. It would be difficult to argue that these covariates should be included at the same time as the data used to identify the patient subgroups as a means of examining the predictive validity of the latent class classification. Another problem with the 1-step approach is that simultaneously including a large number of covariates in a single step may be too cumbersome due to the sparseness of the frequencies and the increased computation time [38]. However, the 3-step approach is not without its own challenges because it does not account for the classification errors that may systematically underestimate the association between potential predictors and class membership [23, 37, 39]. In our study, we used a modified 3-step BCH method that uses a weighting procedure to account for this possible classification error, which has been shown to provide less biased estimates [22].

While acknowledging many possible methods and modelling specifications that would be difficult to verify outside of the artificial context of simulation studies, articulating the methods and the rationale underlying each modelling decision should be given more standing when using GMM because such decisions could potentially change the results (and therefore their interpretation). From this perspective, our contribution to the field is in illustrating how researchers could bring more transparency to the method of analysis and in highlighting issues related to model building with regards to using GMM.

## Conclusions

The use of GMM has the potential to provide valuable information to identify and assess differences in health trajectories, which could lead to tailored subgroup-specific clinical interventions. We found that meaningful longitudinal analyses of PRO data stored in clinical registries need to align closely with patient-centred approaches by accounting for unobserved subgroups of patients and the variability in the frequency and timing of relevant measurement occurrences. However, in analysing these PRO data using GMM, further modelling issues need to be considered (e.g., the selection of appropriate time metrics, specifying growth parameters, and when to include covariates to predict class membership) that could potentially lead to different conclusions.

## Data Availability

The data that support the findings of this study are available from Population Data BC but restrictions apply to the availability of these data, which were used under the license of the current study, and so are not publicly available. Data are however available from the authors upon reasonable request and with permission of Population Data BC and relevant Data Stewards.

## Abbreviations

PRO: Patient-reported outcomes
AF: Atrial fibrillation
AFEQT: Atrial Fibrillation Effect on QualiTy-of-Life Questionnaire
ICD: International Classification of Diseases
FIML: Full information maximum likelihood
ANOVA: Analysis of Variance
MLM: Multilevel model
LGM: Latent growth model
GMM: Growth mixture model
LCGA: Latent class growth analysis
AIC: Akaike’s information criterion
BIC: Bayesian information criterion
SABIC: Sample size adjusted BIC
